# Clinical characteristics and prognostic factors of adult hemophagocytic syndrome patients: a retrospective study of increasing awareness of a disease from a single-center in China

**DOI:** 10.1186/s13023-015-0224-y

**Published:** 2015-02-15

**Authors:** Fei Li, Yijun Yang, Fengyan Jin, Casey Dehoedt, Jia Rao, Yulan Zhou, Pu Li, Ganping Yang, Min Wang, Rongyan Zhang, Ye Yang

**Affiliations:** Department of Hematology, The First Affiliated Hospital of Nanchang University, NanChang, China; Department of Urology, Tulane University Health Science Center, New Orleans, LA USA; Tumor Center, The First Hospital of Jilin University, Changchun, China; Department of Pathology, University of Iowa Carver College of Medicine, Iowa City, USA; State Key Laboratory of Natural Medicines, School of Life Science and Technology, China Pharmaceutical University, Nanjing, China

**Keywords:** Hemophagocytic lymphohistiocytosis (HLH), Clinical characteristics, Laboratory tests, Prognosis

## Abstract

**Background:**

Hemophagocytic lymphohistiocytosis (HLH) is a relatively rare but life-threatening disease with confusing clinical manifestations, rapidly deteriorating health, high morbidity and mortality.

**Methods:**

To improve the recognition as well as understanding of this disorder, we analyzed clinical characteristics and prognostic factors from 85 adult patients diagnosed with HLH in our hospital from April 2005 to June 2014.

**Results:**

Patients with HLH displayed variable clinical markers across a wide spectrum. These included fever and hyperferritinemia (100%), elevated lactate dehydrogenase (LDH) (98.8%), two or three cytopenia (92.2%), splenomegaly (72.9%), hypofibrinogenemia (69.4%), hypertriglyceridemia (64.7%), hemophagocytosis (51.7%), and hepatomegaly (24.7%). Patients with active Epstien-Barr Virus (EBV) infection had a median overall survival (OS) of 65 days. Those displaying malignancy had very poor survival (median OS: 40 days). However, patients in rheumatic and non-EBV infection groups had relatively superior prognosis (not reached). Univariate analysis showed that Fibrinogen (Fbg) <1.5 g/L, platelet number (PLT) <40 × 10^9^/L and LDH ≥1000 U/L were factors that negatively affected survival (*P* = 0.004, 0.000, 0.002). Multivariate analysis showed that PLT <40 × 10^9^/L was the independent adverse factor (HR = 0.350, 95% CI: 0.145-0.844, *P* = 0.019).

**Conclusions:**

HLH had very complex clinical manifestations and high death rate. Patients with active EBV infection, malignancy, Fbg <1.5 g/L, PLT <40 × 10^9^/L and LDH ≥1000 U/L had high risk of death as well as inferior survival, and these patients require systemic targeted treatments as early as possible.

## Background

Hemophagocytic Syndrome (HPS), also known as hemophagocytic lymphohistiocytosis (HLH), is a potentially life-threatening immune system disorder characterized by cytokine storm and overwhelming inflammation [1]. Cytotoxic cells and macrophages cause multiorgan damage, hemophagocytosis, and severe systemic inflammation [2]. The clinical presentations of HLH were generally prolonged fever, hepatosplenomegaly, cytopenia, hypertriglyceridemia, hyperferritinemia, and hemophagocytosis in bone marrow, liver, spleen or lymph nodes [3]. Patients with primary HLH usually have a family history of the disease, or known underlying genetic defects which predispose them to the disease [4]. Secondary HLH is usually caused by some etiologies including infections, autoimmune diseases, malignancies, acquired immune deficiency, as well as iatrogenic immune suppression and organ or stem cell transplantation [5,6].

HLH is a relatively rare disease but has garnered increased attention over the past ten years. More than 1500 publications on this topic have appeared since 2004. Moreover, HLH was once considered a children’s disease (<14 years old). However, cases are being reported with increasing frequency in adults [7]. Patients with HLH present with a wide spectrum of clinical manifestations, rapidly deteriorating conditions, as well as considerable morbidity and mortality.

To improve the recognition and understanding of this disorder in elderly HLH patients (>14 years old) in China, we analyzed data from 85 patients diagnosed at the First Affiliated Hospital of Nanchang University from April 2005 to June 2014. We demonstrated the variable clinical spectrums of these patients, and determined the poor prognosis factors related to HLH patient’s survival.

## Methods

### Patients and tests

This study was approved by the institutional review board of the First Affiliated Hospital of Nanchang University, according to the guidelines of the 1996 Helsinki Declaration. From April 2005 to June 2014, a total of 85 adult HLH patients were admitted to the First Affiliated Hospital of Nanchang University in Nanchang, China. Review of the patient’s medical records fulfilled the requirement for written informed consent in regards to the study. Patients younger than 14 years old were excluded from the study.

Retrospective evaluation included assessment of underlying diseases, clinical manifestations, laboratory findings, treatments, and outcomes. Laboratory findings included peripheral blood examination, alanine aminotransferase (ALT), aspartate aminotransferase (AST), albumin (ALB), bilirubin (BIL), serum creatinine (Scr), triglycerides (TG), lactate dehydrogenase (LDH), prothrombin time (APTT), thrombin time (TT), fibrinogen (Fbg), serum ferritin (SF), blood immunology, virology, bacteriology, bone marrow morphology, flow cytometry, bone marrow tissue immunohistochemical staining, imaging tests for liver, spleen, lymph nodes including B ultrasound examination and computerized tomography (CT). Epstein-Barr virus (EBV) and cytomegalovirus (CMV) infection were diagnosed based on the detection of IgM antibodies or high levels of EBV or CMV DNA [8]. Disseminated intravascular coagulation (DIC) was defined as prolonged prothrombin time, hypofibrinogenemia, or increased fibrinogen degradation products [9].

### Diagnostic criteria of HLH

All patients were diagnosed according to revised diagnostic criteria guideline of the HLH-2004 protocol [3]. The diagnosis of HLH can be established if either A or B is fulfilled: (A) genetic defect consistent with HLH including *PRF1*, *UNC13D*, *STX11*, *STXBP2*, *XIAP*, *AP3B1*, *Rab27a*, *LYST* or *SH2D1A*. (B) ≥ 5 out of 8 clinical and laboratory criteria fulfilled: 1) fever: >38.5°C for ≥7 days, 2) splenomegaly, 3) cytopenias affecting at least two of three lineages, 4) hypertriglyceridaemia (≥3 mmol/L) or hypofibrinogenaemia (<1.5 g/L), 5) hyperferritinaemia (≥500 μg/L), 6) soluble CD25 (soluble interleukin 2 receptor) >2,400 U/mL, 7) hemophagocytosis in bone marrow, spleen, or lymph nodes, 8) low or absent NK-cell cytotoxicity. Only 2 out of 85 patients underwent genetic testing but with negative results. The tests for soluble CD25 levels and NK cell activity were not available at our institution at the time of this study.

### Treatment regimens

All 85 patients received the following treatment regimens: 1) etoposide ± glucocorticoid ± cyclosporine, 2) glucocorticoid ± antibiotics, 3) ECHOP (etoposide, glucocorticoid, vincristine, cyclophosphamide, and adriamycin) [3,10-12]. None of the patients in this study received hematopoietic cell transplantation after chemotherapy due to poor treatment response or aggressive conditions. EBV-related HLH patients were also treated with ganciclovir. Patients with sepsis were administered with sensitive antibiotics. Patients with liver lesion were given drugs protecting liver function. Patients with anemia or coagulopathy were transfused with red cells, fibrinogen or fresh frozen plasma.

### Statistical analysis

Overall survival (OS) was calculated from the date of treatment initiation till death. Survival curves were estimated by the Kaplan–Meier method, differences between curves were tested for statistical significance using the log-rank test. Categorical variables were compared using nonparametric tests and the Pearson’s Chi-square test. Multivariate analysis was performed using the cox-regression method. A *P* value of < 0.05 was considered statistically significant. All data analyses were performed using the statistical software SPSS version 20.0.

## Results

### Clinical and laboratory examination characteristics in 85 patients with HLH

**Figure 1:**
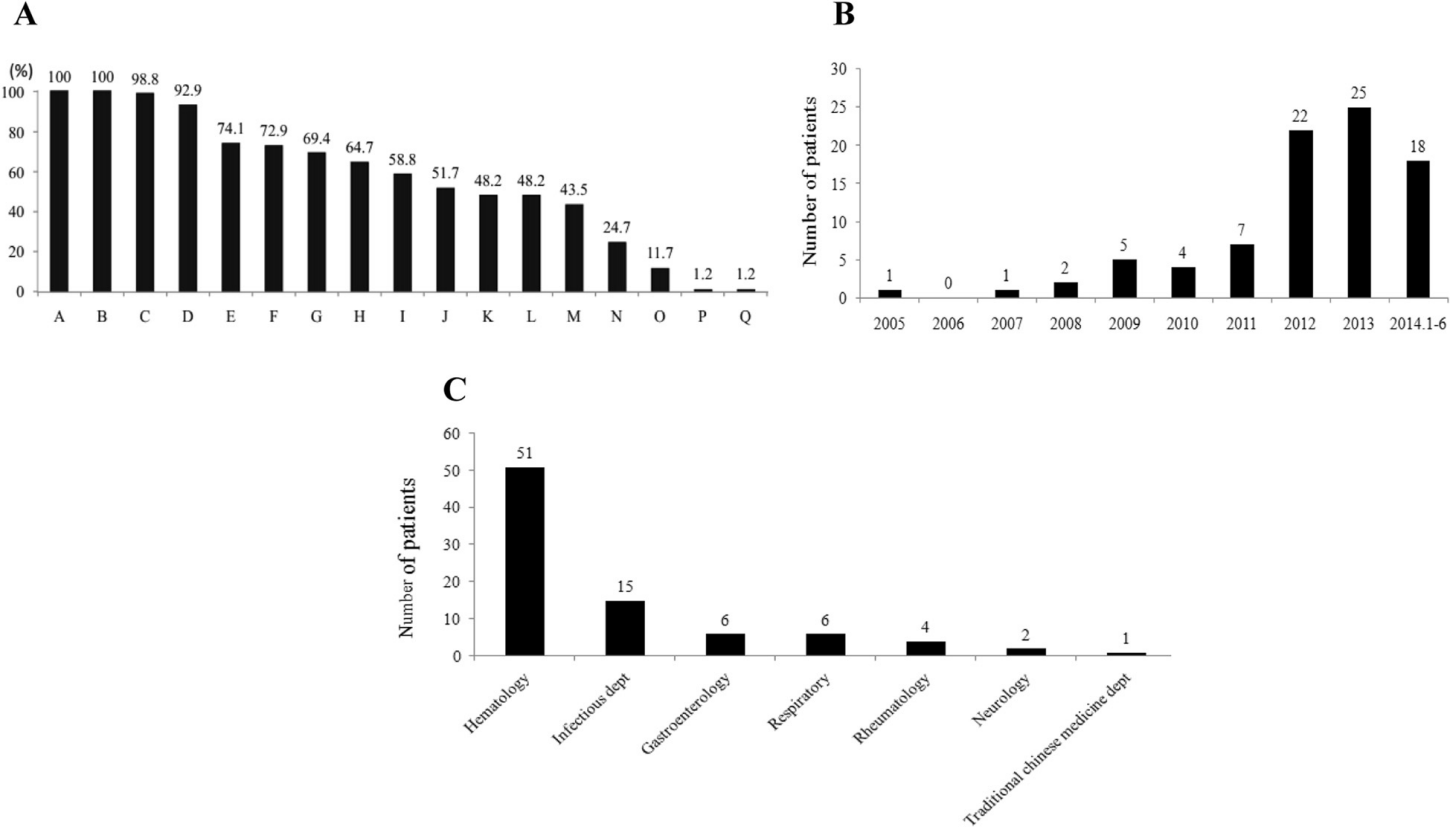
**The characteristics of 85 adult HLH patients. A**. The percentages of clinical symptoms, signs and laboratory examinations in 85 HLH patients. A: fever, B: ferritin ≥500 g/L, C: LDH ≥220 U/L, D: cytopenia in two or more lineages, E: GPT >40 U/L, F: splenomegaly, G: hypofibrinogenaemia, H: hypertriglyceridemia, I: KPTT prolonged 10 S, J: Hemophagocytosis in bone marrow, K: PT prolonged 3S, L: jaundice, M: Lymphadenopathy, N: hepatomegaly, O: renal impairment, P: skin rash, Q: epilepsy. **B**. The distribution of diagnostic time in 85 HLH patients. **C**. The initially hospitalized departments in 85 HLH patients.

A total of 85 patients (64 male, 21 female) fulfilled the HLH diagnostic criteria. The median age was 44 years old (range, 15–72 yr). The median count of blood cells were as follows: WBC 1.66 (0.1-14.1) × 10^9^/L, Hb 86 (43–143) g/L, and PLT 32 (1–130) × 10^9^/L. The clinical manifestations and laboratory findings varied significantly (Figure 1A). The initial symptom of 100% of the patients was fever with temperature fluctuating from 38.5 to 41°C. The duration of fever from its onset to diagnosis ranged from 4 to 180 days. 92.9% of patients had cytopenia (thrombocytopenia <100 × 10^9^/L, leucopenia <4.0 × 10^9^/L, or anemia <90 g/L) in 2 or more cell lines. 25.9% of patients had two cytopenia, 67.1% had three cytopenia, and only 7.1% of patients had sole thrombocytopenia. 72.9% of patients had splenomegaly protruding 1–12 cm below the left costal margin. 24.7% patients had hepatomegaly projecting 1–5 cm below the right costal margin. 43.5% of patients had superficial or deep lymphadenopathy. Bone marrow smears or biopsy revealed hemophagocytosis in 51.8% of patients. One patient had skin rash.

The most common biochemical indication was high LDH (>220 U/L, 98.8%), followed by low hypoalbuminemia (<30 g/L, 89.4%), elevated levels of alanine aminotransferase (>40 U/L, 74.1%), and elevated levels of aspartate aminotransferase (>40 U/L, 88.2%). The median level of ALT was 78 (14–674) U/L lower than the median level of AST (129, range from 17–1489 U/L) (*P* < 0.001), which suggested chronic hepatitis-like performance. Hyperbilirubinemia (total bilirubin level >34 μmol/L) and renal impairment (the level of creatinine >120 μmol/L) accounted for 48.2% and 11.7% of patients, respectively. 58.8% and 48.2% of the patients had prolonged levels of KPTT (10 seconds greater than control) and PT (3 seconds greater than control). One patient presented with epilepsy as a symptom, no other obvious neurological symptoms were exhibited in the sample population.

In seven years from 2005.1 to 2011.12, we only diagnosed 20 HLH patients. With the increased understanding of HLH diagnosis, 47 patients were diagnosed from 2012.1 to 2013.12. In six months from 2014.1 to 2014.6, a total of 18 adult HLH patients were diagnosed. The distribution of diagnostic time in 85 HLH patients is shown in Figure 1B.

60% of patients were initially hospitalized in the department of hematology for symptoms such as fever, cytopenia, hypofibrinogenaemia, coagulopathy. The other 34 patients were sporadically hospitalized in various departments; infections (15), gastroenterology (6), respiratory (6), rheumatology (4), neurology (2), or traditional Chinese medicine department (1) (Figure 1C).

### Underlying diseases in 85 patients with HLH

Table 1 presents the distribution of the underlying diseases in 85 patients with HLH. The majority of underlying diseases in HLH patients were infectious (n = 29, 34.1%), followed by unidentified causes (n = 27, 31.8%), malignancies (n = 23, 27.1%) and autoimmune disorders (n = 6, 7.0%). In 29 HLH patients with infectious diseases, viral infection was the most common cause, 10 (34.4%) cases were caused by EBV infection, two cases were caused by CMV infection and one case was caused by HIV infection. 23 patients in the population displayed malignancy-associated HLH (M-HLH). Of these patients two had aggressive NK cell leukemia, three had B-cell lymphoma, seventeen had T and NK cell lymphoma, and one patient presented a T-lymphoblastic leukemia. 11 of the 23 patients diagnosed with M-HLH simultaneously carried EBV infections. Specific underlying disorder(s) were not determined in 27 of the cases due to limitations in diagnostic technologies and the rapid clinical deterioration of the patients. Of the 27 patients, 14 proceeded with an aggressive disease course, showing lymphadenopathy, splenomegaly, or cells in bone marrow leading to suspicion of lymphoma. However no pathological evidence of lymphoma was found.

**Table 1:** **The distribution of underlying diseases in 85 patients with HLH**

**Underlying diseases**	**No. of patients** **%**
**Infection**	29 (34.1%)
*Viral infection*	
EBV	10
CMV	2
Herpes virus	1
HIV	1
*Sepsis*	
Staphylococcus aureus	2
Klebsiella pneumoniae	4
aeruginosa	1
*Pulmonary infection*	
Candida albicans	1
Klebsiella pneumoniae	3
Unknown	2
*Intestinal infection*	
Escherichia coli	2
**Autoimmune disorders**	6 (7.1%)
Rheumatoid arthritis	2
Systemic lupus erythematosus	2
Adult Still’s disease	2
**Malignancy**	23 (27.1%)
Aggressive NK cell leukemia	2
B-cell lymphoma	3
T&NK cell lymphoma	17
T lymphoblastic leukemia	1
**Unknown causes**	27 (31.8%)

### Survival analysis

**Figure 2:**
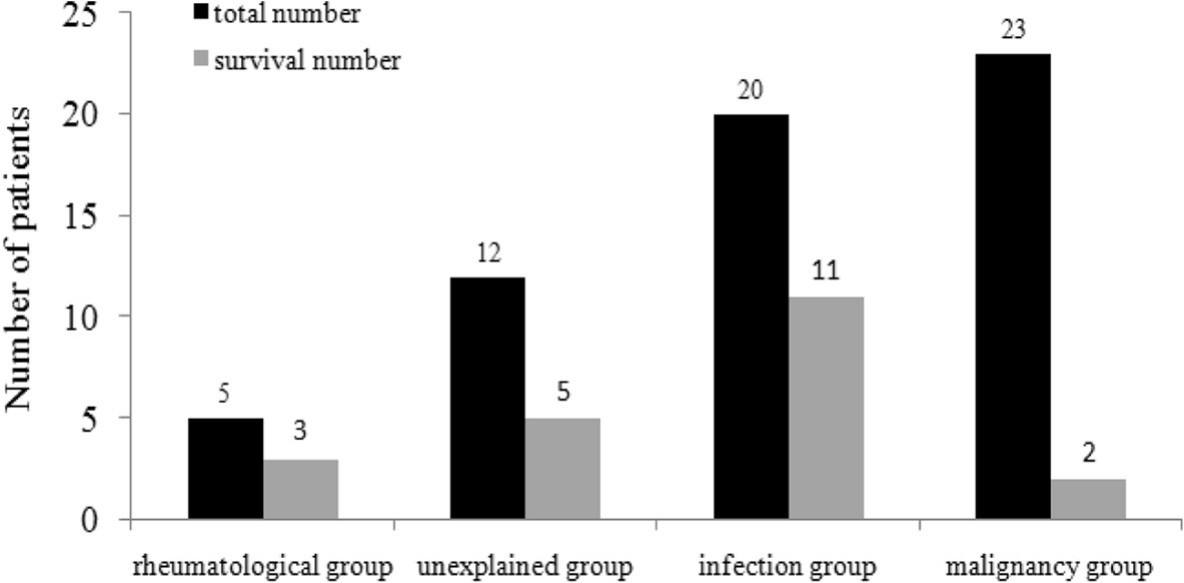
**The number of survive patients in different subgroups.**

To ascertain which specific clinical or laboratory factors at diagnosis predicted prognosis of HLH patients, we analyzed the survival data of the 85 HLH patients. Follow-up data were obtained from 60 patients, 23 patients in the malignancy group, 12 patients in the unknown causes group, 20 patients in the infection group and 5 patients in the rheumatic disease group. Follow up data was lost for 25 of the patients due to poor contact information. Analysis of survivability in different groups is shown in Figure 2. The causes of death were primarily organ hemorrhage and coagulopathy including DIC, intracranial hemorrhage, gastrointestinal hemorrhage, septic shock and multiple organ dysfunction syndrome.

**Figure 3:**
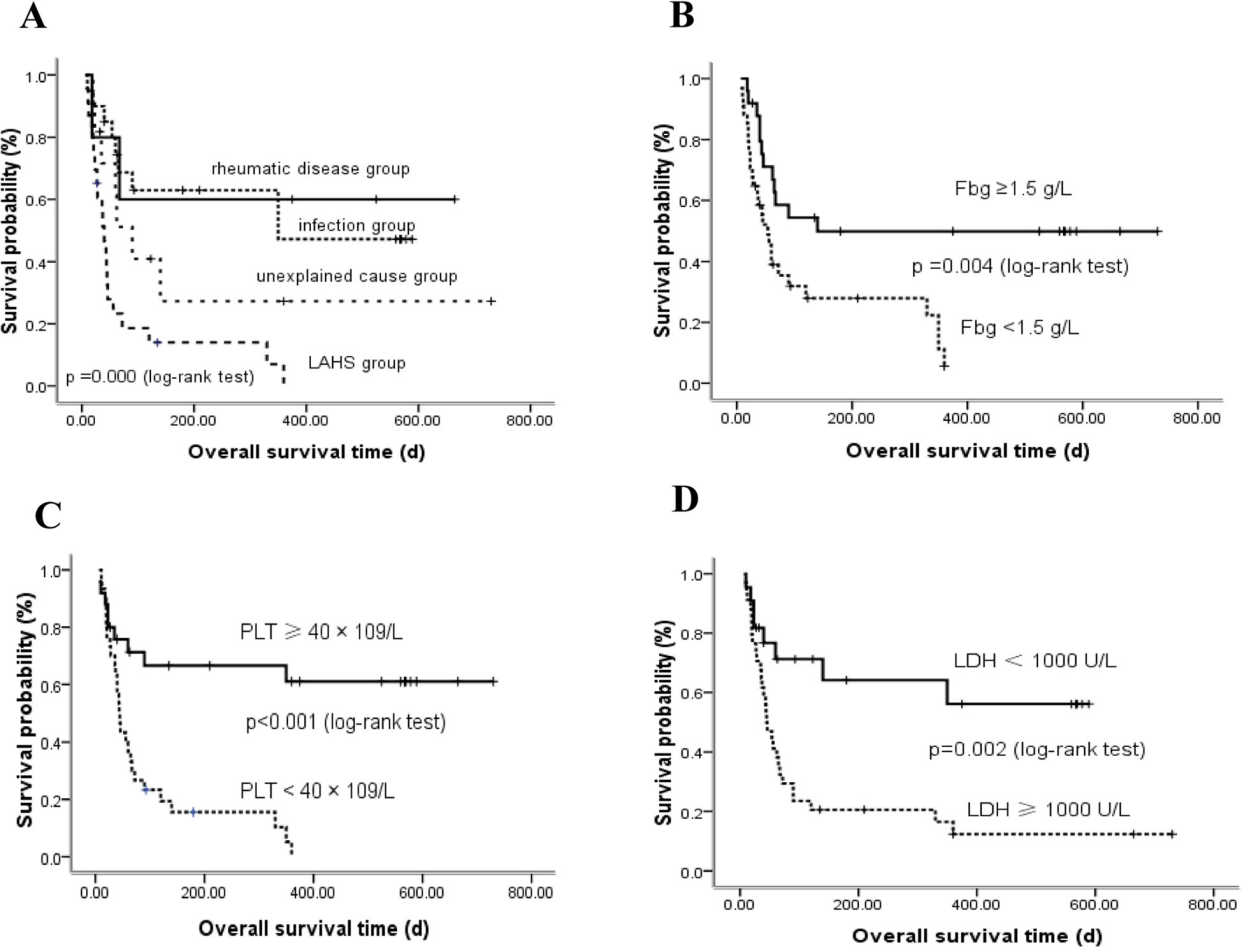
**Survival analysis in 85 cases and different subgroups. A**. Kaplan-Meier survival in 85 HLH patients. **B**. The survival curves of patients in the fibrinogen (Fbg) subgroup. **C**. The survival curves of patients in the platelet (PLT) subgroup. **D**. The survival curves of patients in the lactate dehydrogenase(LDH) subgroup.

OS of patients in the rheumatic disease group was not reached. The median OS of patients in the infection group was 350 days, among the nine dead patients, five patients had EBV infection, one patient had EBV and CMV infection, one patient had HIV infection, and two patients had sepsis. The OS of patients in the unexplained cause group was 90 days. The OS of M-HLH patients was the shortest with 40d (Figure 3A), 78.3% (18/23) of patients died within three months. We analyzed the correlation of EBV infection and the prognosis of patients in infection and malignancy groups, the result showed patients with active EBV infection had shorter survival than patients without EBV infection in infection group (65 days vs. not reached, *P* = 0.021), but no difference in malignancy group.

To find some indicators to predict the risk of death in HLH patients, we compared the difference of various laboratory indicators between survived and dead patients. There was no difference among many of the indicators including white blood cells, hemoglobin, serum ferritin, transaminases, bilirubin and triglycerides (*P* > 0.05). However, the levels of platelet count and fibrinogen in the dead group were significantly lower than those of the patients in the survival group (*P* = 0.008 and 0.045, respectively). Also of note, lactate dehydrogenase was higher in the dead group as compared to the survival group (*P* = 0.005). The comparisons of laboratory parameters between patients in dead and survival groups are shown in Table 2.

**Table 2:** **The comparison of laboratory parameters between patients in death and survival groups**

**Group**	**Number**	**PLT (×10** ^**9**^ **/L)**	**Fbg (g/L)**	**LDH (U/L)**
Death group	39	27.0 (5 ~ 113)	1.02 (0.64 ~ 4.26)	1260 (234 ~ 8560)
Survival group	21	50 (18 ~ 173)	1.51 (0.68 ~ 5.78)	786 (213 ~ 1901)
*P* value		0.008	0.045	0.005

We further analyzed the potential risk factors that might affect the prognosis of HLH. Univariate analysis suggested that patients with Fbg <1.5 g/L (median OS, 54 ± 8.6 d vs. 140 days, *P* = 0.004, Figure 3B), PLT<40 × 10^9^/L (median OS, 44 ± 4.1 days vs. not reached, *P* < 0.001, Figure 3C) or LDH ≥1000 U/L (median OS, 45 ± 8.0 days vs. not reached, *P* = 0.002, Figure 3D) generally had a worse outcome than the control group (Table 3). Multivariate analysis identified PLT<40 × 10^9^/L was only independent predictor of poor OS (HR = 0.350, 95% CI: 0.145-0.844, *P* = 0.019).

**Table 3:** **The comparison of OS time among different subgroup patients**

**Group**	Number*	**OS (d)**	**P value**
Fbg			
<1.5 g/L	34	54 ± 8.6	0.004
≥1.5 g/L	25	140	
LDH			
<1000 U/L	22	Not reached	0.002
≥1000 U/L	34	45 ± 8.0	
PLT			
<40 × 10^9^ /L	30	44 ± 4.1	0.000
≥40 × 10 ^9^/L	25	Not reached	

*Some patients lacked several results due to the retrospective property of this study.

## Discussion

HLH is an infrequent but potentially life-threatening hyperinflammation syndrome, which is caused by a range of inherited or secondary factors. The onset of F-HLH is usually within the first 6 months of life, however, patients with a later onset, even up to adulthood have also been reported [13,14]. Five different forms of F-HLH have so far been described and four genes (*PFR1*, *UNC13D*, *STX11*, *STXBP2*) have been identified. Secondary HLH (sHLH) can develop at any age and is usually caused by underlying conditions such as infection, autoimmune/rheumatologic, malignant conditions et cetera [1]. Currently, data as reported in the literature is not precise enough to predict the incidence of HLH, although single-center studies have reported the incidence to be 0.12/100,000 per year in familial-HLH (F-HLH) patients and 0.36/100,000 per year in M-HLH patients [15]. HLH in young children/infants, mainly occurring F-HLH with a genetic defect, developed similar symptoms to patients from our study with a couple exceptions. Similar symptoms included fever (91%), splenomegaly (81%), hepatomegaly (81%), and lymph node enlargement (43%), however, the incidence of neurologic symptoms (47%), and Rash (43%) might be higher in F-HLH and neurologic symptoms occurred earlier than other symptoms according to Zhang’s report [14]. This study has focused on the clinical characteristics and prognostic factors of adult HLH patients to improve the recognition and understanding of this disorder.

First manifestation of HLH could resemble common infections, malignancies, fever of unknown origin, or autoimmune disorders [16]. The clinical features are thought to be due to hypercytokinemia, such as tumor necrosis factor TNF-α, interferon (IFN)-γ, interleukin (IL)-10, IL-12, and IL-18 released by highly activated lymphocytes and macrophages [17]. Typical clinical findings are prolonged fever, insensitivity to antibiotic therapy, and hepatosplenomegaly. Laboratory findings include cytopenias, usually beginning with thrombocytopenia evolving into severe pancytopenia, hyperferritinemia, elevated transaminases, hypofibrinogenemia, DIC, hypertriglyceridemia, hypoalbuminemia, and hyponatremia [18]. Our data also showed that patients with HLH had variable clinical spectrums as mentioned above (Figure 1A). Due to the confusing clinical symptoms, 49% of the patients were initially misdiagnosed and hospitalized in infectious, gastroenterology, respiratory, or other departments for fever, liver lesion, dizziness, or gastrointestinal bleeding. Due to our increasing recognition based on clinical and laboratory report and the improvements in pathological diagnosis techniques in our hospital, the numbers of patients diagnosed as HLH raised significantly in our institution. In addition, since deficient NK-cells activity and sCD25 are hallmark of HLH and exhibited in major HLH patients [19], the lacking these two tests may veil the real HLH patients numbers diagnosed in our institution. Therefore, we infer that the incidence of HLH occur more commonly than it appeared at least in Jiangxi state.

In this study, we queried the most common form of HLH in adults, infection associated HLH. Infectious triggers include viruses (for example, EBV, cytomegalovirus, HIV), bacteria (for example, mycobacteria), and fungi (for example, candida, cryptococcus) [20]. Among viral infections, EBV is undoubtedly the major cause of HLH. Published data have shown that very high levels of proinflammatory cytokines are associated with EBV-related HLH among Asians [21]. In our current study, 33.8% of patients had EBV infection which is consistent with the result of a Japanese study (33.3%) [22]. Interestingly, a study showed a quarter of male patients with EBV-associated HLH may have mutations in the *SH2D1A* gene, which is traditionally associated with X-linked lymphoproliferative syndrome (XLPS) immunodeficient to EBV [23]. In our data, male HLH patients presented with EBV infections in 36.1% of cases. In contrast, female patients had a rate of 23.8%, moreover, the incidence of HLH was higher in male patients than in females (3:1 ratio). Determining if the higher HLH incidence in males was related to EBV infections will need to be confirmed in a future study. The clinical outcome heterogeneity of patients with EBV-HLH is striking, ranging from self-limiting to aggressive and fatal. Some results indicated that patients with active EBV-HLH or high EBV genome copy numbers had poor prognosis [24,25]. In the infection subgroup, we found patients with active EBV infection had shorter survival than non-EBV infected patients (65d vs. not reached, *P* = 0.021) but there was no significance in the M-HLH subgroup. The high mortality rate among patients with EBV-HLH is due to, at least in part, delays in diagnosis that result from the similarity between its initial clinical presentation and common infective and inflammatory conditions. Of note, improving clinical outcomes and diagnosing patients with refractory EBV-HLH is still challenging. However, outcomes have improved significantly with the use of rituximab, etoposide, and HCT for these patients [26].

We continued to investigate malignant manifestation in HLH patients. M-HLH develops most frequently in patients with T and NK-cell lymphoma, the strong association between the T-cell and NK-cell lymphomas and HLH was reported in several previous studies [27,28]. T and NK-cell associated HLH has also been shown to have the lowest five year survival rate (12%) [14]. Proper T and NK-cell function is required for clearance of antigenic stimuli and termination of the inflammatory response. Aberrant T-cell and NK-cell activation results in excessive cytokine production and sustained macrophage activation. Clonality studies have shown that a significant number of patients with EBV-associated HLH have a clonal proliferation of T cells [29,30]. M-HLH can also occur in the course of other hematological malignancies (e.g., B-cell lymphoma, Hodgkin’s lymphoma, acute and chronic leukemias) [31]. Ishii et al. [32] reported that in Japan the causes of 18% of M-HLH cases were cancers other than lymphoma such as acute myeloid leukemia and myelodysplastic syndromes. Our results were in accordance with the above, 82.6% of the patients in this subset (N = 23) had T and NK cell lymphoma/leukemia, three patients had B-cell lymphoma, and one patient had T lymphoblastic leukemia. 47.8% (11/23) of patients simultaneously had EBV infection.

HLH patients have a high mortality rate, which is partially caused by a lack of clinical suspicion and unavailability of genetic as well as other molecular studies in most developing countries. Therefore, it is necessary to establish effective pretreatment markers to predict the high-risk subsets in this disorder. In this study, we further explored the prognostic factors related to HLH survival in clinics. Our results showed patients with M-HLH had the worst prognosis (median OS: 40 days), and patients in unexplained cause group also had poor outcomes (median OS: 90 days. This may have been caused in part by an inability to diagnose lymphoma in patients that were suspected of having it due to lack of evidence.

To exclude the interference of different hospitalized divisions, we compared the survival time among patients from various departments. The results revealed that patients initially hospitalized in hematology had the shortest survival time (median OS, 45 days), followed by patients hospitalized in other departments (median OS, 350 days) and infectious department (median OS, not reached) (*P* = 0.006) (data not shown). This result is consistent with our conclusion that M-HLH and EBV infection are associated with poor survival. This is due to the fact that there were 19 patients with lymphoma and 10 with EBV infection admitted to the hematology department. Also, there was no survival difference between patients diagnosed before and after 2011 (data not shown). Intriguingly, we found patients with Fbg <1.5 g/L, PLT <40 × 10^9^/L, or LDH ≥1000 U/L had shorter OS by using univariate analysis which is consistent with our previous study in lymphoma - associated HLH (LAHS) [33]. Furthermore, multivariate analysis demonstrated PLT <40 × 10^9^/L was the sole predictor of poor OS, which is consistent with the reports from Wang Z et al. [34,35]. The mechanism under which PLT is operating remains elusive. We inferred that PLT change might be a more direct consequence of cytokine storm and hyperinflammation compared to Fbg and LDH.

HLH is an aggressive and incurable disease. Even with treatment and haematopoietic stem cell transplant (HSCT), the 5-year survival probability ranges from 45% to 75%, but the survival was 0% without HSCT treatment [12]. Due to the aggressive life-threatening implications of HLH, effective treatments including therapies that target activated macrophages/histiocytes (etoposide, steroids, high-dose IVIgG) and/or activated T cells (steroids, cyclosporine A, antithymocyte globulins) [36] should be instituted promptly, followed by HSCT. The survival of patients diagnosed in a study conducted at South Carolina University with EBV-HLH was significantly improved when etoposide treatment started within 4 weeks of diagnosis [37]. Besides, treatment on coexisting infections, identification of other potential triggers of HLH is also necessary. Patients who do not respond to steroids require management with aggressive combination chemotherapy, followed by HSCT. In our group, the median OS is 60 days despite the implementation of treatments such as etoposide, glucocorticoid and cyclosporine. The reason for poor survival in these patients may be as follows, the unavailability of some high specificity indicator tests [38,39] such as soluble CD25 levels and NK cell activity in our institution resulted in delayed diagnoses and treatment of HLH patients which led to end-organ failure and death. Moreover, high-dose chemotherapy drugs including etoposide and allo-HCT regimens were not applied in these patients. These treatments could considerably improve survival for patients with refractory EBV-associated HLH and lymphoma [12].

In this article, our results echo clinical studies completed elsewhere that both T/NK-cell abnormalities as well as EBV viral infection play an important role in the disease progression [27,30,32]. This study attempted to evaluate effective markers for diagnoses across a wider spectrum of cases and long follow-up time (up to 9 years). Studies in the past usually focused on identifying sole clinical marker to this disease [37]. With a better idea of confidence and significance, we disclose the importance of factors like malignance disease and EBV infection et al. simultaneously on the sHLH based on a comprehensive interpretation of solid sample. However, other studies showed some different prognostic factors such as the high level of serum ferritin to our data [35]. More studies would need to be performed to verify these results.

## Conclusions

Taken together, our data revealed HLH adult patients had a variable clinical spectrum as well as underlying diseases. Patients with active EBV infection and lymphoma had poor survival. Patients with Fbg <1.5 g/L, PLT <40 × 10^9^/L and LDH ≥1000 U/L had high risk of death as well as inferior survival, and effective treatment like high-dosage chemotherapy combined with HSCT might be critical to these patients.

## References

[1] Chandrakasan S, Filipovich AH (2013). Hemophagocytic lymphohistiocytosis: advances in pathophysiology, diagnosis, and treatment. J Pediatr.

[2] Larroche C (2012). Hemophagocytic lymphohistiocytosis in adults: diagnosis and treatment. Joint Bone Spine.

[3] Henter JI, Horne A, Arico M, Egeler RM, Filipovich AH, Imashuku S, Ladisch S, McClain K, Webb D, Winiarski J, Janka G (2007). HLH-2004: diagnostic and therapeutic guidelines for hemophagocytic lymphohistiocytosis. Pediatr Blood Cancer.

[4] Canna SW, Behrens EM (2012). Not all hemophagocytes are created equally: appreciating the heterogeneity of the hemophagocytic syndromes. Curr Opin Rheumatol.

[5] Fukaya S, Yasuda S, Hashimoto T, Oku K, Kataoka H, Horita T, Atsumi T, Koike T (2008). Clinical features of haemophagocytic syndrome in patients with systemic autoimmune diseases: analysis of 30 cases. Rheumatology (Oxford).

[6] Kuzmanova SI (2005). The macrophage activation syndrome: a new entity, a potentially fatal complication of rheumatic disorders. Folia Med.

[7] Janka GE, Lehmberg K (2014). Hemophagocytic syndromes–an update. Blood Rev.

[8] Sakamoto Y, Mariya Y, Kubo K (2012). Quantification of Epstein-Barr virus DNA is helpful for evaluation of chronic active Epstein-Barr virus infection. Tohoku J Exp Med.

[9] Kaneko T, Wada H (2011). Diagnostic criteria and laboratory tests for disseminated intravascular coagulation. J Clin Exp Hematop.

[10] Henter J-I, Samuelsson-Horne A, Aricò M, Egeler RM, Elinder G, Filipovich AH, Gadner H, Imashuku S, Komp D, Ladisch S (2002). Treatment of hemophagocytic lymphohistiocytosis with HLH-94 immunochemotherapy and bone marrow transplantation. Blood.

[11] Strout MP, Seropian S, Berliner N (2010). Alemtuzumab as a bridge to allogeneic SCT in atypical hemophagocytic lymphohistiocytosis. Nat Rev Clin Oncol.

[12] Trottestam H, Horne A, Arico M, Egeler RM, Filipovich AH, Gadner H, Imashuku S, Ladisch S, Webb D, Janka G, Henter JI (2011). Chemoimmunotherapy for hemophagocytic lymphohistiocytosis: long-term results of the HLH-94 treatment protocol. Blood.

[13] Nagafuji K, Nonami A, Kumano T, Kikushige Y, Yoshimoto G, Takenaka K, Shimoda K, Ohga S, Yasukawa M, Horiuchi H (2007). Perforin gene mutations in adult-onset hemophagocytic lymphohistiocytosis. Haematologica.

[14] Zhang K, Jordan MB, Marsh RA, Johnson JA, Kissell D, Meller J, Villanueva J, Risma KA, Wei Q, Klein PS, Filipovich AH (2011). Hypomorphic mutations in PRF1, MUNC13-4, and STXBP2 are associated with adult-onset familial HLH. Blood.

[15] Machaczka M, Vaktnas J, Klimkowska M, Hagglund H (2011). Malignancy-associated hemophagocytic lymphohistiocytosis in adults: a retrospective population-based analysis from a single center. Leuk Lymphoma.

[16] Janka GE (2007). Hemophagocytic syndromes. Blood Rev.

[17] Larroche C, Mouthon L (2004). Pathogenesis of hemophagocytic syndrome (HPS). Autoimmun Rev.

[18] Arico M, Janka G, Fischer A, Henter JI, Blanche S, Elinder G, Martinetti M, Rusca MP (1996). Hemophagocytic lymphohistiocytosis. Report of 122 children from the International Registry: FHL Study Group of the Histiocyte Society. Leukemia.

[19] Usmani GN, Woda BA, Newburger PE (2013). Advances in understanding the pathogenesis of HLH. Br J Haematol.

[20] Bode SF, Lehmberg K, Maul-Pavicic A, Vraetz T, Janka G, Stadt UZ, Ehl S (2012). Recent advances in the diagnosis and treatment of hemophagocytic lymphohistiocytosis. Arthritis Res Ther.

[21] Filipovich AH (2005). Life-threatening hemophagocytic syndromes: current outcomes with hematopoietic stem cell transplantation. Pediatr Transplant.

[22] Tabata YS, Teramura T, Kuriyama K, Yagi T, Todo S, Sawada T, Imashuku S (2000). Molecular analysis of latent membrane protein 1 in patients with Epstein-Barr virus-associated hemophagocytic lymphohistiocytosis in Japan. Leuk Lymphoma.

[23] George MR (2014). Hemophagocytic lymphohistiocytosis: review of etiologies and management. J Blood Med.

[24] Imashuku S, Teramura T, Tauchi H, Ishida Y, Otoh Y, Sawada M, Tanaka H, Watanabe A, Tabata Y, Morimoto A (2004). Longitudinal follow-up of patients with Epstein-Barr virus-associated hemophagocytic lymphohistiocytosis. Haematologica.

[25] Kunitomi A, Kimura H, Ito Y, Naitoh K, Noda N, Iida H, Sao H (2011). Unrelated bone marrow transplantation induced long-term remission in a patient with life-threatening Epstein-Barr virus-associated hemophagocytic lymphohistiocytosis. J Clin Exp Hematop.

[26] Ohga S, Kudo K, Ishii E, Honjo S, Morimoto A, Osugi Y, Sawada A, Inoue M, Tabuchi K, Suzuki N (2010). Hematopoietic stem cell transplantation for familial hemophagocytic lymphohistiocytosis and Epstein-Barr virus-associated hemophagocytic lymphohistiocytosis in Japan. Pediatr Blood Cancer.

[27] Lay JD, Tsao CJ, Chen JY, Kadin ME, Su IJ (1997). Upregulation of tumor necrosis factor-alpha gene by Epstein-Barr virus and activation of macrophages in Epstein-Barr virus-infected T cells in the pathogenesis of hemophagocytic syndrome. J Clin Invest.

[28] Tong H, Ren Y, Liu H, Xiao F, Mai W, Meng H, Qian W, Huang J, Mao L, Tong Y (2008). Clinical characteristics of T-cell lymphoma associated with hemophagocytic syndrome: comparison of T-cell lymphoma with and without hemophagocytic syndrome. Leuk Lymphoma.

[29] Chan LC, Srivastava G, Pittaluga S, Kwong YL, Liu HW, Yuen HL (1992). Detection of clonal Epstein-Barr virus in malignant proliferation of peripheral blood CD3+ CD8+ T cells. Leukemia.

[30] Kanegane H, Bhatia K, Gutierrez M, Kaneda H, Wada T, Yachie A, Seki H, Arai T, Kagimoto S, Okazaki M (1998). A syndrome of peripheral blood T-cell infection with Epstein-Barr virus (EBV) followed by EBV-positive T-cell lymphoma. Blood.

[31] Machaczka M, Nahi H, Karbach H, Klimkowska M, Hagglund H (2012). Successful treatment of recurrent malignancy-associated hemophagocytic lymphohistiocytosis with a modified HLH-94 immunochemotherapy and allogeneic stem cell transplantation. Med Oncol.

[32] Ishii E, Ohga S, Imashuku S, Yasukawa M, Tsuda H, Miura I, Yamamoto K, Horiuchi H, Takada K, Ohshima K (2007). Nationwide survey of hemophagocytic lymphohistiocytosis in Japan. Int J Hematol.

[33] Li F, Li P, Zhang R, Yang G, Ji D, Huang X, Xu Q, Wei Y, Rao J, Huang R, Chen G (2014). Identification of clinical features of lymphoma-associated hemophagocytic syndrome (LAHS): an analysis of 69 patients with hemophagocytic syndrome from a single-center in central region of China. Med Oncol.

[34] Weng Y, Chen N, Han Y, Xing Y, Li J (2014). Clinical and laboratory characteristics of severe fever with thrombocytopenia syndrome in Chinese patients. Braz J Infect Dis.

[35] Huang W, Wang Y, Wang J, Zhang J, Wu L, Li S, Tang R, Zeng X, Chen J, Pei R, Wang Z (2014). [Clinical characteristics of 192 adult hemophagocytic lymphohistiocytosis]. Zhonghua Xue Ye Xue Za Zhi.

[36] Filipovich AH. Hemophagocytic lymphohistiocytosis (HLH) and related disorders. Hematol Am Soc Hematol Educ Program. 2009:127–31. https://www.ncbi.nlm.nih.gov/pubmed/20008190. 10.1182/asheducation-2009.1.127 20008190

[37] Shabbir M, Lucas J, Lazarchick J, Shirai K (2011). Secondary hemophagocytic syndrome in adults: a case series of 18 patients in a single institution and a review of literature. Hematol Oncol.

[38] Switala JR, Hendricks M, Davidson A (2012). Serum ferritin is a cost-effective laboratory marker for hemophagocytic lymphohistiocytosis in the developing world. J Pediatr Hematol Oncol.

[39] Lehmberg K, McClain KL, Janka GE, Allen CE (2014). Determination of an appropriate cut-off value for ferritin in the diagnosis of hemophagocytic lymphohistiocytosis. Pediatr Blood Cancer.

